# 5-aminolevulinic acid-mediated photodynamic therapy and its strain-dependent combined effect with antibiotics on *Staphylococcus aureus* biofilm

**DOI:** 10.1371/journal.pone.0174627

**Published:** 2017-03-30

**Authors:** Qing-Zhao Zhang, Ke-Qing Zhao, Yang Wu, Xian-Hui Li, Chen Yang, Li-Min Guo, Chun-Hong Liu, Di Qu, Chun-Quan Zheng

**Affiliations:** 1 Department of Otorhinolaryngology-Head and Neck Surgery, Eye & ENT Hospital, School of Shanghai Medicine, Fudan University, Shanghai, PR China; 2 Key Laboratory of Medical Molecular Virology of Ministries of Education and Health, School of Basic Medical Science and Institutes of Biomedical Sciences, Shanghai Medical College of Fudan University, Shanghai, China; 3 Department of Otolaryngology, The Third Affiliated Hospital of Wenzhou Medical University, Wenzhou, China; 4 Department of Otolaryngology, Ruijin Hospital, School of medicine, Shanghai Jiao Tong University, Shanghai, China; 5 Department of Clinical Laboratory, Eye and ENT Hospital, Fudan University, Shanghai, China; Massachusetts General Hospital, UNITED STATES

## Abstract

*Staphylococcus aureus* (*S*. *aureus*) is hard to be eradicated, not only due to the emergence of antibiotic resistant strains but also because of its ability to form biofilm. Antibiotics are the major approach to treating biofilm infections, but their effects are unsatisfactory. One of the potential alternative treatments for controlling biofilm infections is photodynamic therapy (PDT), which requires the administration of photosensitizer, followed by light activation. 5-aminolevulinic acid (ALA), a natural photosensitizer prodrug, presents favorable characteristics, such as easy penetration and rapid clearance. These advantages enable ALA-based PDT (ALA-PDT) to be well-tolerated by patients and it can be repeatedly applied without cumulative toxicity or serious side effects. ALA-PDT has been proven to be an effective treatment for multidrug resistant pathogens; however, the study of its effect on *S*. *aureus* biofilm is limited. Here, we established our PDT system based on the utilization of ALA and a light-emitting diode, and we tested the effect of ALA-PDT on *S*. *aureus* biofilm as well as the combined effect of ALA-PDT and antibiotics on *S*. *aureus* biofilm. Our results showed that ALA-PDT has a strong antibacterial effect on *S*. *aureus* biofilm, which was confirmed by the confocal laser scanning microscope. We also found that lethal photosensitization occurred predominantly in the upper layer of the biofilm, while the residual live bacteria were located in the lower layer of the biofilm. In addition, the improved bactericidal effect was observed in the combined treatment group but in a strain-dependent manner. Our results suggest that ALA-PDT is a potential alternative approach for future clinical use to treat *S*. *aureus* biofilm-associated infections, and some patients may benefit from the combined treatment of ALA-PDT and antibiotics, but drug sensitivity testing should be performed in advance.

## Introduction

*Staphylococcus aureus* (*S*. *aureus*) is a major cause of hospital-acquired and community-acquired pathogenic bacteria [1]. Its ability to form biofilm makes it difficult to be eradicated by the human immune system and traditional treatments, namely antibiotics [2]. It has become imperative to develop alternative approaches to manage biofilm infections.

Photodynamic therapy (PDT), an alternative to antibiotics for the treatment of bacterial infection, uses photosensitizers activated by the light of an appropriate wavelength. The activation of the photosensitizers leads to the production of reactive oxygen species (ROS), which is lethal to target cells [3].

5-aminolevulinic acid (ALA) is a prodrug that is converted to a natural photosensitizer, protoporphyrin IX (PpIX), in target cells [4]. ALA presents several favorable characteristics. First, ALA is a natural intermediate in the heme biosynthetic pathway that can be rapidly cleared from the target cells [5]. Second, ALA is a prodrug of the real photosensitizer PpIX and is small enough to penetrate the matrix around cells and accumulate in the target cells [6]. Lastly, ALA’s photodynamic effect is restricted to superficial lesions (1–2 mm) because of the limited penetration of the light source [7]. These characteristics may shorten light avoidance time, reduce tissue damage, and improve the efficacy of PDT, all of which ensure the safety and efficacy of PDT in clinical application.

The effect of PDT on *S*. *aureus* biofilm has been investigated with different photosensitizers, such as malachite green, methylene blue, and sinoporphyrin sodium [8,9,10]; however, the antimicrobial effect of ALA-PDT and its combined effect with other approaches on *S*. *aureus* biofilm is still extremely limited. In this study, we tested the antibacterial effect of ALA-PDT and its combined effect with antibiotics on *S*. *aureus* biofilm, and our results showed that ALA-PDT has a strong bactericidal effect against *S*. *aureus* biofilms. In addition, the sequential combination of ALA-PDT and antibiotics led to improved bactericidal efficacy on *S*. *aureus* biofilm in a strain-dependent way.

## Materials and methods

### Clinical *S*. *aureus* strain collection

Approval was obtained from the Fudan University Institutional Review Board to enroll adult patients who met the guidelines for chronic rhinosinusitis (CRS) with or without nasal polyps [11]. All participants included in this study were provided written informed consent. Detailed information about the patients' age, gender, etc. was recorded. Swabs were obtained from the middle meatus of the CRS patients during endoscopic surgery performed from January–June 2015 at the Department of Otorhinolaryngology-Head and Neck Surgery, Eye and ENT Hospital of Fudan University. The swabs were sent to the clinical microbiology laboratory for microbiologic characterization and antibiotic sensitivity testing to select methicillin sensitive *staphylococcus aureus* (MSSA) and methicillin resistant *staphylococcus aureus* (MRSA) strains. The isolates were stored in 35% Tryptone Soya Broth (TSB) at -80°C for the following experiments.

### Biofilm assay

Biofilm formation of *S*. *aureus* was detected by a microtiter plate assay as previously described [12]. Briefly, bacterial strains were inoculated in a Tryptone Soya Agar (TSA) plate for 24 h at 37°C. A single colony was chosen and inoculated in 5 ml of TSB for 12 h at 37°C. Then the bacterial suspension was diluted to 1:200 with 5 ml of TSB and incubated another 12 h at 37°C to obtain bacteria in an optimal state. These bacteria were collected by centrifugation, and re-suspended in PBS to a final concentration of 2.0×10^9^ colony forming unit (CFU)/ml. Then, the bacterial suspension was diluted to 1:200 in TSB (1x10^7^ CFU/ml). We placed 200 μL of bacterial suspension per well in a 96-well plate and incubated at 37°C for 24 h, followed by washing with PBS 3 times to remove all non-adherent cells. We added 100 μL of methanol to each well to fix the attached bacteria at room temperature for 12 min, and then they were removed and air-dried. Each well was filled with 100 μl of 2% crystal violet and incubated at room temperature for 10 min. The wells were dumped, rinsed with running tap water until the water was clear, and then incubated with 100 μL of 10% acetic acid with shaking for 1 h at room temperature. Finally, the optical density (OD) of each well stained with crystal violet was measured at 570 nm using a microtiter-plate reader. (DTX 880 Multimode Detector, Beckman Coulter, USA). The USA300 and USA300 mutant of transposon insertion in the *icaA* gene (*icaA* mutant) were used as positive and negative biofilm-forming controls in the experiment, respectively. The clinical strains were divided into 4 groups according to their biofilm-forming ability as measured by OD_570_. The cutoff OD_570_ value (ODc) was defined as 3 standard deviations above the mean OD_570_ of the negative control and the OD_570_ value of a tested strain was expressed as the average value of 3 replicates. The *S*. *aureus* isolates were divided into the following groups: 1) no biofilm producer (-): OD_570_≤ODc; 2) weak biofilm producer (+): ODc<OD_570_<2xODc; 3) moderate biofilm producer (++): 2xODc<OD_570_≤4xODc; and 4) strong biofilm producer (+++): 4xODc<OD_570_ [13].

### Minimum inhibitory concentration (MIC) assay

Broth micro-dilution testing was used to determine the MICs in accordance with the guidelines provided by the American Clinical and Laboratory Standards Institute [14]. A range of different concentrations (0.125–128 μg/mL) of netilmicin, vancomycin, and cefaclor in cation-adjusted Mueller Hinton broth was prepared and dispensed into a microtiter plate (50 μL/well) and 50 μL of bacteria (10^6^ CFU/mL) was then added into each well, resulting in the final concentrations of these antibiotics, which ranged from 0.0625–64 μg/ml. After 20 h of incubation at 37°C, the MIC value was determined as the lowest concentration of the antibiotics to completely inhibit bacterial growth as observed by the naked eye. The *S*. *aureus* (ATCC 29213) strain was used as the quality control strain.

### Effects of antibiotics on biofilm

The 96 wells were gently washed with PBS 3 times and supplanted with the indicated concentration of netilmicin, vancomycin or cefaclor, respectively, from 0 to 500 μg/ml to test the effects of netilmicin, vancomycin, or cefaclor on the biofilms, followed by the biofilm formation. Then, the plates were incubated for 24 h at 37°C. After that, the wells were carefully washed with PBS 3 times and the bacteria were scratched and collected in Eppendorf (EP) tubes. They were centrifuged at 6000 g for 10 min at 4°C, then the solution was removed and 1 ml of 0.25% pancreatin enzymes was added to suspend the bacteria at 37°C [15]. After 1.5 h of incubation, the bacteria were centrifuged again and diluted serially by PBS. We then added 5 μL of bacterial suspension to the TSA plate. After 16 h of incubation of the TSA at 37°C, the number of CFU/ml was calculated to determine the bacterial viability of the biofilm.

### PDT study design

**Fig 1:**
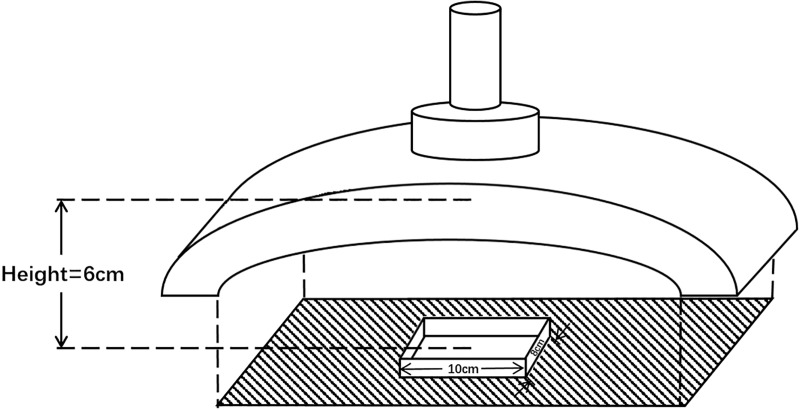
Schematic drawing of the experimental set-up for light delivery. The experimental region was restricted to the central area (10cm x 8cm) under the light source. Distance from the peak of the light source to the experimental region was fixed at 6cm.

We used ALA (Fudan Zhangjiang Bio-Pharm, Shanghai, China) and a light-emitting diode (LED) array (Wuhan Yage Optic and Electronic Technique CO., Wuhan, China) with a major wavelength of 633±10nm as the photosensitizer and the light source, respectively, in our study. The distance from the peak of the light source to the well was fixed at 6.0 cm to keep the light energy delivered to the biofilm equal in all of the experiments. The central illumination area (10 cm x 8 cm) was used as the experimental region (Fig 1). The room temperature was maintained at 25°C during all of the experiments.

Biofilms were incubated with serial dilutions of ALA in darkness for varying time durations or exposed to serial doses of light without ALA to test the toxicity of ALA and light.

In the PDT experiment, the biofilms were incubated with the indicated concentration of ALA in darkness and then were irradiated by LED with the indicated dose. After that, the number of CFU/ml was calculated to determine the bacterial viability of the biofilm as described above.

### Combined treatments

To test the combined effect of ALA-PDT and antibiotics for all of the biofilm-forming strains, followed by ALA-PDT, the 96 wells were gently washed with PBS 3 times and supplanted with the indicated concentration of netilmicin, vancomycin or cefaclor, respectively. Then, the plates were incubated for 24 h at 37°C in darkness. After that, the bacterial viability (CFU/ml) was calculated.

### Observation of *S*. *aureus* biofilms by CLSM

The effect of ALA-PDT on the *S*. *aureus* biofilms was evaluated by LIVE/DEAD staining. Briefly, after ALA-PDT irradiation, the biofilms were washed with PBS 3 times, and then stained with 1 uM of SYTO9 and 1 μM of propidium for 20 min. The stained cells were visualized by a Leica TCS SP5 confocal laser-scanning microscope (CLSM) (Leica TCS SP8 Confocal Laser Scanning Platform, Leica Microsystems, Germany) with a 63x 1.4-NA oil immersion objective. Three-dimensional biofilm images were created with IMARIS 7.0 software (Bitplane, USA).

### Data analysis

CFU/ml was log-transformed before statistical analysis. Data are expressed as mean ± standard error. Unpaired two-tailed *t*-test was used for between-group analyses. One-way ANOVA followed by Boneferroni’s post hoc tests were exploited for comparison of 3 or more groups. All analyses were performed using SigmaPlot 13.0 (Systat Software Inc., USA). Two-tailed P values <0.05 were considered to be statistically significant.

## Results

### Characteristics of the clinical staphylococcal isolates

The 45 *S*. *aureus* strains of MSSA (36) and MRSA (9) were collected after microbiologic characterization testing. Of these isolates, 26 (72.2%) were biofilm-negative strains and 10 (27.8%) were biofilm-positive strains in the MSSA group, and 4 (44.4%) were biofilm-negative and 5 (55.6%) were biofilm-positive strains in the MRSA group. In total, 15 biofilm-forming isolates were included in this study (Table 1). The MICs of netilmicin, vancomycin, and cefaclor for the 15 isolates are summarized in Table 2.

**Table 1:** The biofilm-forming abilities of the clinical *S*.*aureus* strains.

	Biofilm-negative^a^ (number(%))	Biofilm-positive^a^ (number(%))		
Strains	-	+	++	+++
MSSA	26(72.2%)	3(8.3%)	3(8.3%)	4(11.1%)
MRSA	4(44.4%)	1(11.1%)	2(22.2%)	2(22.2%)

^a^ The *S*.*aureus* strains were further classified into four groups according to their abilities to form biofilms as measured by OD_570_. The cutoff OD value (ODc) was 0.31. The four groups were as follows: no biofilm producer (-), OD_570_≤0.31; weak biofilm producer (+), 0.31<OD_570_≤0.62; moderate biofilm producer (++),0.62<OD_570_≤1.24; and strong biofilm producer (+++) 1.24<OD_570_.

**Table 2:** MICs of antibiotics for the clinical *S*. *aureus* strains.

MSSA ID	Netilmicin	Vancomycin	Cefaclor	MRSA ID	Netilmicin	Vancomycin	Cefaclor
*S*.*aureus* 29213	0.25	1	1	1718^+++^	1	0.5	4
129^+++^	0.5	0.5	2	1213^+++^	0.5	1	4
125^+++^	2	1	4	1310^+^	0.5	1	2
991^+++^	0.125	0.5	2	1281^++^	1	1	2
1165^+++^	0.5	1	0.5	672^++^	0.5	1	4
1171^+^	0.125	1	0.5				
1203^++^	0.5	1	1				
165^++^	0.25	0.5	1				
175^++^	0.5	1	1				
234^+^	1	0.5	0.5				
365^+^	0.5	1	0.5				

The unit of the antibiotics is μg/mL

^+^weak biofilm producer

^++^ moderate biofilm producer

^+++^ strong biofilm producer.

### Parameters of ALA and light irradiation

**Fig 2:**
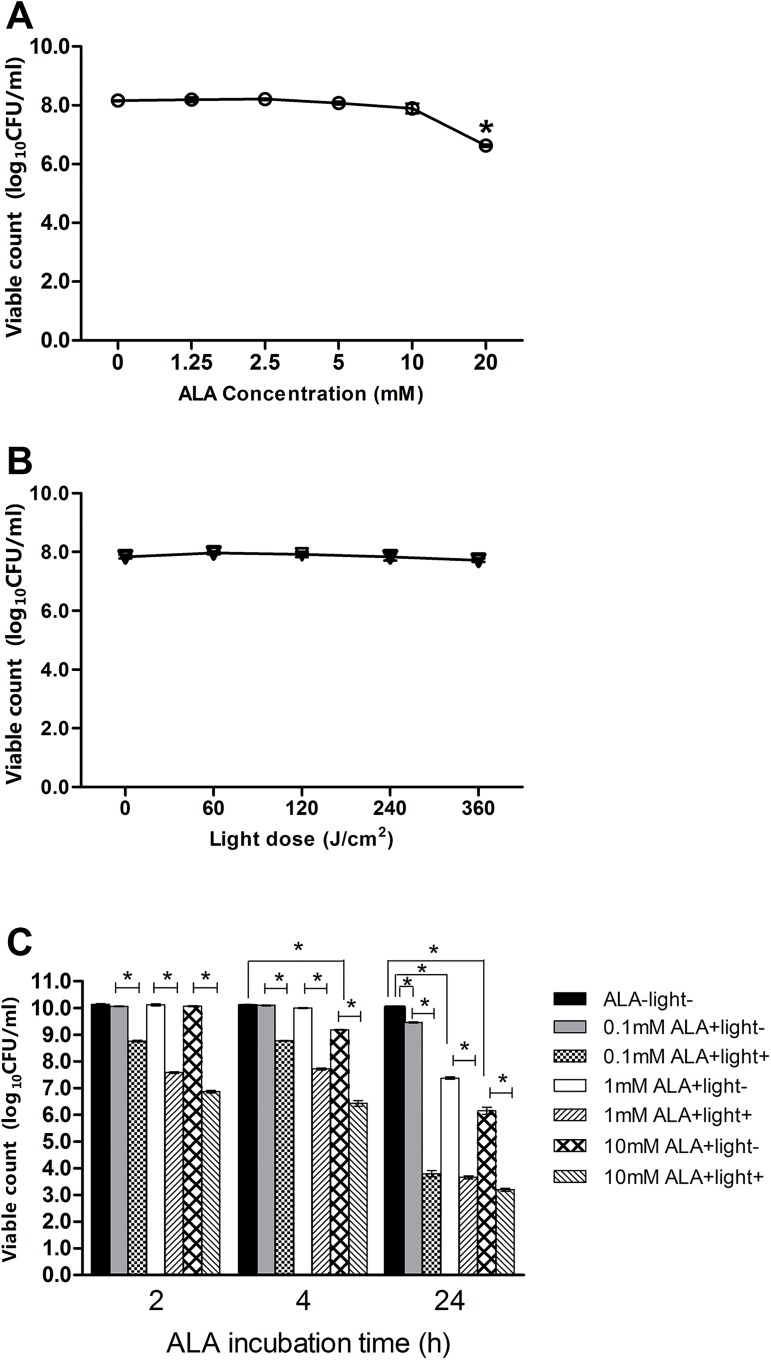
The toxicity of ALA and light irradiation and bactericidal effect of ALA-PDT with different parameters. (A). Biofilms were incubated with serial dilutions of ALA in dark for 2h. A significant reduction of log_10_CFU/ml was observed at 20 mM of ALA group (6.63±0.04) compared with 0 mM of ALA group (8.16±0.01) (n = 3,*P<0.05). (B). No toxicity was found in any of the light irradiation groups. (C). The bactericidal effect of ALA-PDT with the combinations of different concentrations of ALA and different durations of incubation (n = 3,*P<0.05).

We performed tests to exclude the toxicity of ALA or light irradiation to *S*. *aureus* biofilm, respectively, to determine the parameters of the dose of the ALA and light irradiation used in our study. No toxicity of ALA was observed at or less than 10 mM of ALA with 2 h of incubation in darkness (Fig 2A). In addition, no CFU/ml reduction was found in all of the light irradiation groups we tested (Fig 2B). However, toxicity was found in the 4 h of incubation group with 10 mM of ALA and in the 24 h of incubation group with 0.1 mM, 1 mM and 10 mM groups (Fig 2C). We chose 10 mM of ALA with 2 h of incubation followed by 360 J/cm^2^ light irradiation as the photodynamic parameters, because no toxicity was found in their control group (2 h of incubation with 10 mM of ALA), and their bactericidal effect was stronger than the ones in the groups incubated with 0.1 mM and 1 mM of ALA for 4 h followed by light irradiation (Fig 2C).

### Inactivation of *S*. *aureus* biofilms by ALA-PDT

**Fig 3:**
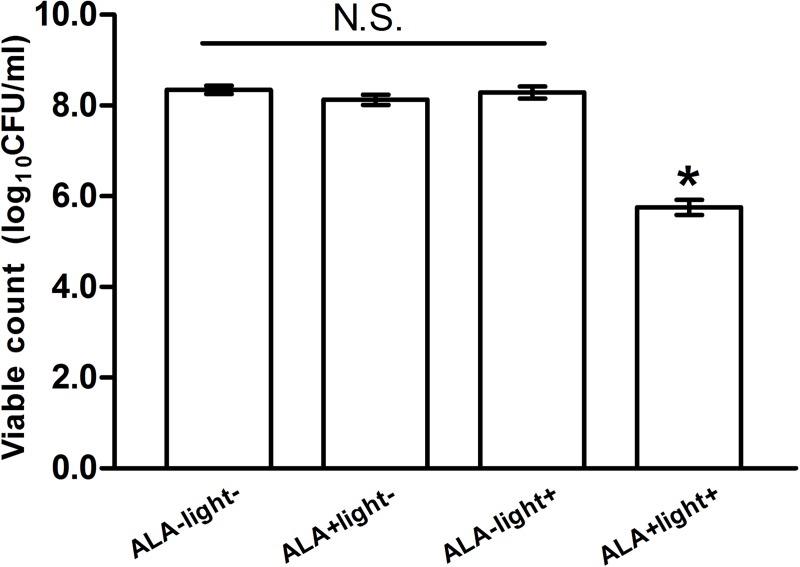
ALA-PDT inactivates *S*.*aureus* biofilm. After ALA-PDT treatment the log_10_CFU count was decreased from 8.35±0.09 (ALA-light- group, n = 15) to 5.75±0.17 (ALA+light+ group, n = 15). No significant difference was found among the three control groups (ALA-light- group: 8.35±0.09, ALA+light- group: 8.13±0.11, and ALA-light+ group: 8.29±0.14, n = 15). N.S.: not significant. *P<0.05: ALA-PDT group vs. controls.

**Fig 4:**
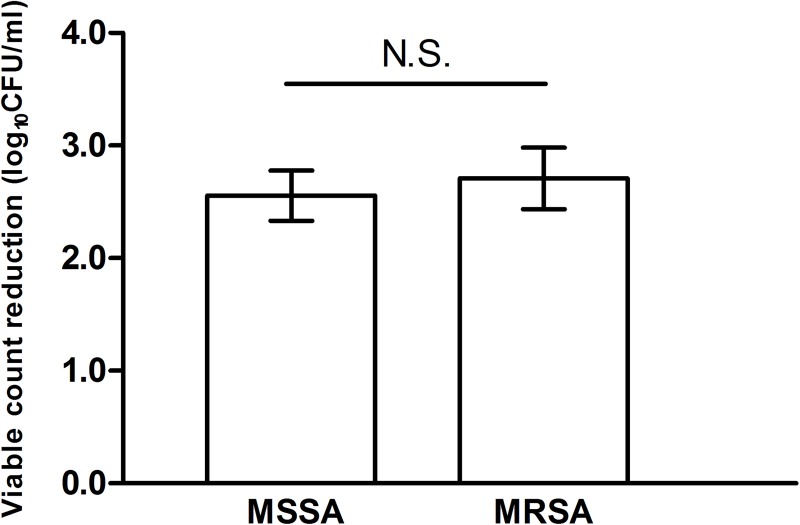
Reduction of bacterial viability after ALA-PDT treatment. No difference of the reduction of viable bacteria was found between the MSSA (2.56±0.22, n = 10) and MRSA group (2.71±0.27, n = 5) after ALA-PDT treatment. N.S.: not significant.

No difference in the viability of the biofilms was found among the 3 controls (ALA-light-:8.35±0.09, ALA+light-:8.13±0.11, and ALA-light+:8.29±0.14, n = 15). However, compared with the control groups, the viability of the biofilm was significantly decreased after ALA-PDT (10 mM of ALA with 360 J/cm^2^ light) treatment (5.75±0.17, n = 15, p<0.01) (Fig 3). Our results also showed that no difference in the reduction of log_10_CFU/ml was found between the MSSA (2.56±0.22, n = 10) and MRSA (2.71±0.27, n = 5) groups after ALA-PDT treatment (Fig 4).

### CLSM results

**Fig 5:**
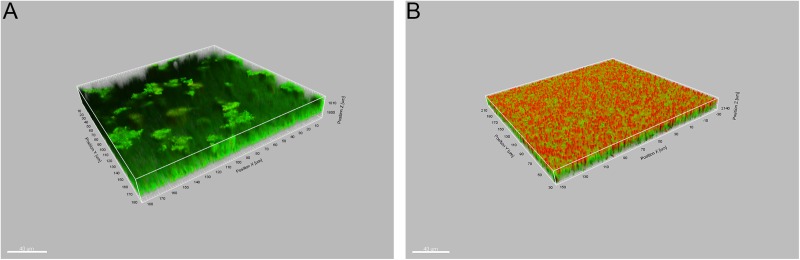
Representative CLSM images of *S*.*aureus* biofilm with LIVE/DEAD staining. (A) Biofilms were treated with 10mM ALA for 2 h in dark. (B) ALA-treated biofilms were exposed to 360J/cm^2^ light irradiation. A 63x 1.4-NA oil immersion objective was used.

To better understand the bactericidal effect of ALA-PDT on the *S*. *aureus* biofilm, the biofilms were visualized by CLSM with LIVE/DEAD BacLight staining. The viable cells were stained with green fluorescence and the dead cells were stained with red fluorescence. The images show that ALA-PDT killed most of the bacteria in the biofilms, while just a few bacteria were dead in the control group. In the ALA-PDT group, the dead bacteria were predominantly distributed in the upper layer, while the live ones were in the lower layer (Fig 5).

### Combined effect of ALA-PDT and antibiotics

**Fig 6:**
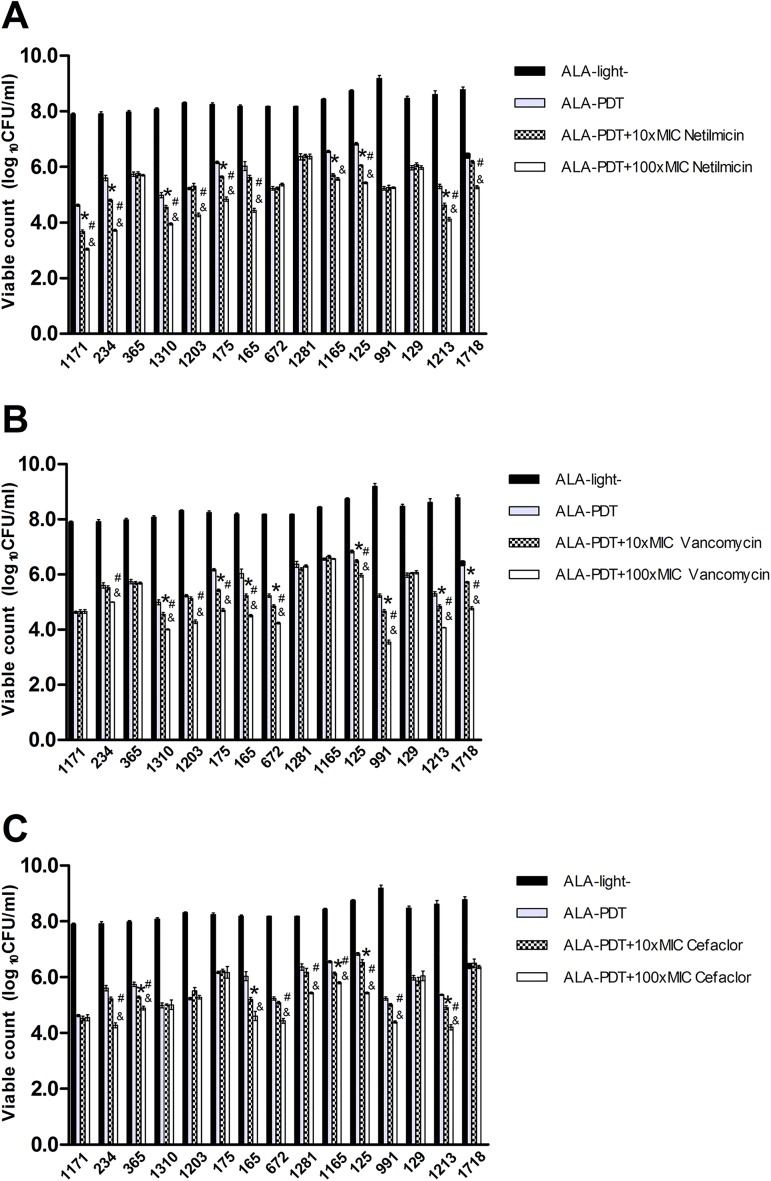
Effect of combined treatment of ALA-PDT and antibiotics on *S*. *aureus* biofilm. Strain-dependent combined effects were observed in Netilmicin (A), Vancomycin (B) and Cefaclor(C) groups. *P<0.05:ALA-PDT+10xMIC group vs. ALA-PDT group, ^&^P<0.05: ALA-PDT+100xMIC group vs. ALA-PDT group, ^#^P<0.05: ALA-PDT+100xMIC group vs. ALA-PDT +10xMIC group.

No bactericidal effect of antibiotics on the *S*. *aureus* biofilm was observed even with the concentration of antibiotics as high as 500 μg/ml (0 μg/ml group: 8.28±0.05, 500μg/ml of netilmicin: 8.24±0.04, 500μg/ml of vancomycin: 8.28±0.06, and 500μg/ml of cefaclor: 8.30±0.05, n = 15). Based on the above results, we chose 10xMIC and 100xMIC as the concentrations (all less than 500 μg/ml) of each antibiotic in testing the combined effect with ALA-PDT. The combined effect was observed in 7/15, 8/15, and 5/15 strains in ALA-PDT combined with 10xMIC netilmicin, ALA-PDT combined with 10xMIC vancomycin, and ALA-PDT combined with 10xMIC cefaclor groups, respectively, and in 10/15, 10/15, and 9/15 strains in ALA-PDT combined with 100xMIC netilmicin, ALA-PDT combined with 100xMIC vancomycin, and ALA-PDT combined with 100xMIC cefaclor groups, respectively (Fig 6).

## Discussion

The administration of exogenous ALA leads to the accumulation of PpIX and induces cellular damage or death after light irradiation by the produced ROS [16]. ALA-PDT has been proven to effectively treat planktonic antibiotic resistant strains [5]. However, contrary to its planktonic counterpart, the sessile structure of the biofilm makes it more difficult to be eradicated. To the best of our knowledge, the studies of ALA-PDT on *S*. *aureus* biofilm are extremely limited [17,18].

Our *in vitro* study clearly showed that ALA-PDT had a strong bactericidal effect on *S*. *aureus* biofilm. More than 99% of the bacteria in the biofilm were killed after the ALA-PDT treatment. This effect was stronger than Barra’s and Li’s studies, in which the largest bactericidal effects of ALA-PDT on the *S*. *aureus* biofilm were about 80% and 95%, respectively [17,18]. We attributed this to the different bacterial sensitivities to ALA-PDT since only one single *S*. *aureus* strain was included in their studies.

The bactericidal effect of PDT on the S. aureus biofilm has also been studied by other teams with different photosensitizers, such as malachite green, methylene blue, and sinoporphyrin sodium [8,9,10]. Our results indicated that ALA-PDT is an effective approach to treat *S*. *aureus* biofilm, which may extend the choice of photosensitizer for PDT in the future. Furthermore, we included multiple clinical strains in this study, which enabled us to compare the bactericidal effect of PDT on MSSA and MRSA biofilms, and to explore the strain-dependent combined effect of ALA-PDT with antibiotics on killing biofilm bacteria. These have still not been well studied to the best of our knowledge.

The effect of ALA-PDT on the *S*. *aureus* biofilm was also revealed by CLSM in our study. The LIVE/DEAD staining can preserve the intact structure of the biofilm, and with this feature, the detailed information, namely the distribution of live and dead cells after PDT, can be observed vividly by CLSM. Our results indicated that most of the cells in the biofilm were inactivated by ALA-PDT; on the contrary, almost all of the cells in the control group were still alive. Interestingly, in the ALA-PDT group, the dead cells were predominantly distributed in the upper layer of the biofilms while some of the lower bacteria were still alive after ALA-PDT. O’Neill et al. observed a similar phenomenon with multi-species biofilms in their study, and explained that this may be due to the low accumulation of photosensitizers in the inner layer, or inability of the light to penetrate into these regions [19].

The increasing prevalence of nosocomial and community-acquired MRSA has presented major health and economic challenges, and MRSA is harder to treat than MSSA due to its extensive resistance to antibiotics. However, the question remains as to whether the biofilm of MRSA is harder to treat than that of MSSA. Our study showed for the first time that the effect of ALA-PDT against the biofilm formed by MRSA and MSSA is not significantly different, which may extend the indications of ALA-PDT in future utilization.

Although the mechanism causing antibiotic resistance of biofilm is still unclear, it is believed that its mechanism is different from that of its planktonic counterparts. It has been noted that the antibiotic resistance of planktonic bacteria depends upon the innate resistance of individual bacteria caused by plasmids, transposons, and mutations [20], while the resistance to the biofilm depends on its multicellular structure [21]. This is based on observations that antibiotic-sensitive bacteria can turn into the resistant phenotype when they form a biofilm [22]. On the contrary, when bacteria are dispersed from biofilm, they will return to the susceptible phenotype again [23]. PDT may break the sessile structure of the biofilm and lead to the recovery of antibiotic sensitivity to the bacteria in the biofilm. Although numerous studies concerning planktonic bacteria described synergistic effect between PDT and antimicrobials, the use of PDT combined with antibiotics was not intensively studied in biofilm [24,25,26,27]. In this study, we combined ALA-PDT with netilmicin, vancomycin, or cefaclor, respectively. It turned out that some strains could be further killed by subsequent antibiotics, while others could not. Our results implied that the sensitivity of antibiotics could be restored by ALA-PDT in a strain-dependent way; this may support the sequential administration of ALA-PDT and antibiotics in some clinical patients.

Two caveats of our study must be acknowledged. First, although our results supported that ALA-PDT has an equal effect on MSSA and MRSA biofilms, this conclusion was based on small experimental groups. Thus, this will need to be confirmed by further studies involving numerous bacterial populations. Second, we cannot explain the strain-dependent effect of sequential applications of ALA-PDT and antibiotics on *S*. *aureus* biofilm. The mechanism behind this phenomenon remains to be uncovered in future studies.

Taken together, our data support the assertion that ALA-PDT is a strong bactericidal approach for *S*. *aureus* biofilm, and the combination of ALA-PDT and netilmicin, vancomycin, or cefaclor could induce combined effect on *S*. *aureus* biofilm in a strain-dependent way. It suggests that ALA-PDT is a promising alternative approach to *S*. *aureus* biofilms, and some patients may benefit from combined treatments, but the drug sensitivity testing should be performed in advance.
